# Evaluation of Emotional Distress in Oncology Palliative Patients: Edmonton Symptom Assessment System (ESAS) and Palliative Outcome Scale (POS)—A Portuguese Cross-Sectional Study

**DOI:** 10.3390/cancers16244232

**Published:** 2024-12-19

**Authors:** Florbela Gonçalves, Margarida Gaudêncio, Ivo Paiva, Francisca Rego, Rui Nunes

**Affiliations:** 1 Faculty of Medicine, University of Porto, 4099-002 Porto, Portugal; mfrego@med.up.pt (F.R.); ruinunes@med.up.pt (R.N.); 2 Internal Medicine and Palliative Care Service—Portuguese Institute of Oncology Francisco Gentil Coimbra, 3000-075 Coimbra, Portugal; 4196@ipocoimbra.min-saude.pt; 3 Health Sciences Research Unit, Nursing, Nursing School of Coimbra, 3000-232 Coimbra, Portugal; ivopaiva@esenfc.pt

**Keywords:** anxiety, cancer, depression, emotional exhaustion, palliative care, palliative medicine, psychological distress

## Abstract

Introduction: Cancer patients in palliative care frequently experience psychological distress, encompassing cognitive, behavioral, and emotional dimensions. This distress can significantly affect their capacity to accept the inevitability of death. Commonly, such distress manifests as sadness, depression, anxiety, and fear, which may culminate in an existential crisis. Objectives: The purpose of this study was to analyze the psychometric properties of the Edmonton Symptom Assessment System (ESAS) and the Palliative Outcome Scale (POS) among hospitalized cancer patients receiving palliative care in a Portuguese palliative care unit. Methods: Data were collected from psychological screening instruments (ESAS and POS) used in oncology patients admitted to an oncology palliative service. The study period spanned from January 2021 to March 2021. Results: The study included a convenience sample of 60 patients diagnosed with advanced cancer who were sequentially admitted to a palliative care unit. The most frequently reported primary oncological diagnoses were gastrointestinal (41.7%) and head/neck (20%) tumors. The majority of the patients were male (*n* = 37; 61.7%), with a median age of 72 years (range: 43–94 years). Key findings from the analysis indicated the following associations: anxiety and self-reported symptoms (higher levels of patient anxiety related to illness or treatment were strongly correlated with increased self-reported anxiety (*p* < 0.01)); emotional sharing and perceived meaning of life (patients who shared their feelings less frequently with family and friends were significantly less likely to find meaning in life (*p* < 0.01) and were more likely to report a poorer self-perception (*p* < 0.05)); understanding the importance of life and self-perception (a diminished understanding of the importance of life was associated with worsened self-perception (*p* < 0.01)); anxiety and depressive symptoms (greater anxiety related to the disease or its treatment correlated with an increased severity of depressive symptoms (*p* < 0.01)). Discussion and conclusion: There are numerous validated instruments available for measuring emotional distress. Among these, the ESAS and POS complement each other as practical and easy-to-use tools for evaluating emotional distress in patients admitted to palliative care settings. Findings revealed that anxiety and concerns related to the disease and its treatment, as measured by the POS, served as predictors of the risk for self-reported anxiety and depression identified by the ESAS. Furthermore, the items within the POS that evaluate the emotional component demonstrated strong internal correlation, supporting their combined use for a comprehensive assessment of this dimension.

## Simple Summary

Patients with advanced cancer often have high levels of emotional suffering, so it is necessary to identify their palliative needs and address them effectively. Although distress is highly prevalent among patients receiving palliative care, research in this area indicates that it remains significantly underdiagnosed and undertreated. The aim of our cross-sectional and correlational study was to analyze the psychometric properties of the Edmonton Symptom Assessment System (ESAS) and the Palliative Outcome Scale (POS) among inpatient cancer patients receiving palliative care in a Portuguese palliative care unit. We confirmed that self-reported anxiety and depression (reported through the ESAS scale) are correlated to emotional questions (reported through the POS), namely, anxiety related to disease, the meaning of life, and sharing feelings. This study showed us that the ESAS and POS complement each other as practical and easy-to-use tools for evaluating emotional distress in the context of palliative care.

## 1. Introduction

Cancer and its trajectory from diagnosis to death are associated with significant distress. According to the National Comprehensive Cancer Network (NCCN), distress is defined as “an unpleasant, multifactorial experience of a psychological (i.e., cognitive, behavioral, emotional), social, spiritual, and/or physical nature that may interfere with a person’s ability to cope effectively with cancer, their physical symptoms and their treatment; extending along a continuum from normal feelings of vulnerability, sadness, and fear of problems that can become disabling, such as depression, anxiety, panic, social isolation and existential and spiritual crisis” [1].

Patients with advanced cancer often have high levels of suffering, so it is necessary to identify their palliative needs and address them effectively. The symptoms that most affect the quality of life of patients in palliative care, often reducing survival, are pain, asthenia, and emotional symptoms such as anxiety and depression [2,3,4].

According to the World Health Organization (WHO), “Palliative care aims to prevent and relieve health related suffering of adults, children and their families facing problems associated with life-threatening illness. It is based on a comprehensive and person-centred approach, addressing physical, psychological, social and spiritual suffering” [5].

Although emotional suffering is highly prevalent among patients receiving palliative care, research in this area indicates that it remains significantly underdiagnosed and undertreated [6,7].

The early identification of psycho-emotional symptoms in patients receiving palliative care is crucial for ensuring timely referral to psychological or psychiatric consultations [8,9].

Numerous validated instruments are described in the literature to measure emotional distress; however, there is no consensus on which one best evaluates it. Examples include the Distress Thermometer (DT) [10], the Hospital Anxiety and Depression Scale (HADS) [11], and the Brief Symptom Inventory (BSI) [12], among others.

These instruments are used to identify and measure the primary symptoms associated with suffering, as well as the presence of depression, anxiety, existential concerns, spirituality, hope, and demoralization [13].

The Emotional Thermometer is considered the instrument of choice for assessing psychological distress; however, it is not always routinely used by palliative care teams. In practice, the Edmonton Symptom Assessment System (ESAS) and the Palliative Outcome Scale (POS) are the most commonly utilized tools for assessing symptoms in these patients [14].

The Edmonton Symptom Assessment Scale (ESAS) [15] is one of the most widely used instruments for identifying and evaluating the intensity of the most common symptoms in patients receiving palliative care [16]. However, it is known that the psychological symptoms assessed by the ESAS—namely, anxiety and depression—are often overestimated by patients, as this scale refers to symptoms experienced over the last week [17,18].

Hearn and Higginson were pioneers in the development of the POS, a scale initially designed to measure outcomes in palliative care [19]. The scale includes 10 items that assess the physical, psychological, social, and spiritual aspects of these patients’ well-being [19]. Items 3, 6, 7, and 8 of the POS are closely related and should be evaluated together, as they pertain specifically to the patient’s psychological well-being [20]. When scores on these items are elevated, it is important to consider the presence of depressive pathology, anxiety disorders, or non-existential spiritual suffering [20]. 

Given the relevant role that instruments such as the ESAS and POS can play in daily practice in palliative care, documenting their validity is equally important. Therefore, the main objective of this study was to analyze the psychometric properties of the Edmonton Symptom Assessment System (ESAS) and the Palliative Outcome Scale (POS).

## 2. Material and Methods

### 2.1. Sample and Design

The authors conducted a cross-sectional, descriptive, and correlational study to assess emotional distress in cancer patients admitted to a Portuguese palliative care unit.

The sample comprised 60 patients who were consecutively admitted between January and March 2021.

The inclusion criteria were defined as adult patients with oncological pathology, receiving palliative care at the hospital, and capable of providing informed, voluntary consent to participate in the study. Exclusion criteria included minors, patients not fluent in Portuguese, those with cognitive deficits, and individuals in the terminal phase of illness.

### 2.2. Ethical Consideration 

Participation in the study was voluntary. Confidentiality and anonymity of participants were assured throughout all stages of the study, with data identified by exclusive code numbers and stored on password-protected laptops, accessible only to the researchers.

### 2.3. Patient and Public Involvement 

Participants were not directly or indirectly involved in the design, implementation, reporting, or dissemination of the study.

### 2.4. Instruments

The authors developed a sociodemographic and clinical questionnaire that included variables such as age, gender, education level, marital status, residence, companions (caregivers), cancer type, and Eastern Cooperative Oncology Group (ECOG) performance status. The ECOG scale is used to assess the functional level of patients, with scores ranging from 0 (fully active) to 4 (completely unable to perform self-care activities) [21].

The ESAS was developed by Bruera in 1991 and is a validated instrument for identifying and measuring the severity of the most common symptoms in patients with advanced cancer. The scale includes several visual and numerical assessments, scored from 0 to 10 (where 0 reflects the absence of the symptom and 10 represents the worst possible intensity), measuring symptoms such as pain, fatigue, nausea, drowsiness, dyspnea, appetite, depression, anxiety, and well-being [15,22]. It is an easy-to-use tool for both patients and healthcare professionals, having been translated and validated in over 20 languages. The scale takes an average of 1 to 2 min to complete, even for cancer patients in palliative care or those with low health literacy [23].

The literature suggests that while the Edmonton Symptom Assessment Scale (ESAS) is not ideal for diagnosing anxiety and depression, it can serve as an effective screening tool in patients with advanced cancer, who often experience multiple symptoms. The use of various instruments to assess each symptom individually can be logistically challenging, making the ESAS a practical and legitimate option for screening. Studies indicate that scores above 2 on the ESAS are sufficiently sensitive to identify the presence of depression and anxiety [24].

The Palliative Outcome Scale (POS) was developed in 1999, originally designed to measure outcomes in palliative care, particularly psychological well-being and quality of care. It is widely used across several European countries and has been translated and validated in multiple languages. The questions focus on the last three days, and the estimated time for completion is approximately 10 min [20].

The Palliative Outcome Scale (POS) includes two versions: one for the patient and one for the clinical staff. It consists of 10 items, each scored from 0 to 4 on a Likert scale, and these items can be considered individually or summed as a total score. The total score ranges from 0 to 40, with 0 indicating the best quality of life and 40 representing the worst. Additionally, the POS includes two open-ended questions related to the patient’s primary concerns [20,25].

The Palliative Outcome Scale (POS) is a multidimensional scale with strong psychometric properties, particularly evident in items 3, 6, 7, and 8. Item 3 also captures family anxiety and should be considered separately. Item 7 (“felt that life was worthwhile”) reflects the spiritual component of the scale [20]. Items 5, 9, and 10 assess the quality of care received, while items 1 and 2 evaluate physical symptoms, including pain and other symptoms such as nausea, cough, and constipation [20].

### 2.5. Statistics

Microsoft^®^ Excel 2016^®^ was used to design the database, and SPSS version 25^®^ was employed for statistical analysis. 

For the statistical analysis, frequencies and percentages were calculated for categorical variables, while medians with interquartile ranges were used for quantitative variables. Spearman’s rank correlation coefficient was applied to evaluate correlations between the various variables, with *p* values less than 0.05 considered statistically significant.

## 3. Results

### 3.1. Sample’s Characteristics

During the study period, 60 patients were included. None of the patients declined participation or were excluded. 

The sample’s participants had a median age of 72 years (interquartile range: [58.25; 79]), with the majority being male (61.7%). Most patients were married (81.7%) and were living at home (83.3%). Regarding tumor type, most participants had digestive cancer (41.7%), followed by head and neck cancer (20%), breast cancer (10%), and lung cancer (10%) (Table 1). 

To assess the functional status of the sample, the authors used the Eastern Cooperative Oncology Group (ECOG) performance status scale. This scale evaluates a patient’s level of functioning based on their ability to perform self-care, engage in daily activities, and maintain physical ability [26].

At admission to the palliative care (PC) unit, 6.6% of participants had an ECOG score of 0, indicating they were fully active and able to perform all activities. Moreover, 10% of patients had an ECOG score of 1, meaning they were restricted in physically strenuous activities but able to perform light work. The majority of participants had ECOG scores of 3 and 4, indicating they were capable of only limited self-care or were completely disabled, with severely restricted functionality.

Regarding the global Palliative Outcome Scale (POS) data, participants had a median POS score of 15 (interquartile range: [13; 19]). The Global Distress Score (GDS), a validated subtype of the Edmonton Symptom Assessment Scale (ESAS), is calculated and categorized into three cohorts: high (GDS ≥ 35), moderate (16 ≤ GDS < 35), and low (GDS < 15) [27]. In this sample, the median GDS score was 43 (range: 17 to 62). The majority of participants (70%) had a GDS ≥ 35, indicating a high symptom burden. 

### 3.2. Edmonton Symptom Assessment Scale (ESAS) and Palliative Outcome Scale (POS)—Emotional and Psychological Dimensions

Regarding the ESAS scale, Table 2 presents the descriptive analysis of the psychological dimensions of the scale in the sample.

In terms of depression, the majority of participants reported moderate to severe levels at palliative care (PC) admission (*n* = 50; 83.3%). Specifically, 39 patients (65%) exhibited moderate levels, corresponding to a score between 4 and 6 on the ESAS scale. On the other hand, 11 patients presented severe levels of distress, with scores ranging from 7 to 10. Only 10 patients did not report depression or had mild levels of distress.

For anxiety, the sample exhibited high levels of this symptom, with 61% of participants reporting moderate anxiety and 27.1% experiencing severe anxiety. These findings are consistent with those observed in the depression dimension.

Table 3, on the other hand, presents the descriptive analysis of the psychological dimensions of the POS scale.

Regarding anxiety related to the disease process and treatment, the majority of participants reported feeling worried about this (*n* = 40; 66.7%). Some patients indicated “Sometimes affects my concentration now and then” (*n* = 13; 21.7%), while others responded “Most of the time often affects my concentration” (*n* = 36; 60%).

The majority of patients felt that their life was worthwhile (*n* = 38; 63.3% – responded “Most of the time”) and some indicated “Sometimes” (*n* = 13; 21.7%). No patients considered their life to be not worthwhile. Furthermore, most patients felt good about themselves as individuals (*n* = 51; 85%), with no patients reporting negative feelings about themselves.

Finally, the authors would like to note that the majority of patients (*n* = 49; 81.7%) completed the POS questionnaire independently. The remaining patients were assisted by a family member or friend (*n* = 6; 10%) or by palliative care staff (*n* = 5; 8.3%).

### 3.3. Relationship Between ESAS and POS Emotional Dimensions 

An analysis was conducted to assess the correlation between the emotional dimensions of the ESAS and POS (Table 4).

A significant correlation was found between self-reported anxiety and anxiety related to the disease (as measured by the POS scale) (*p* < 0.01) (cells in blue). Specifically, higher levels of anxiety related to the disease or treatment were associated with increased self-reported anxiety (ESAS).

It was also found that the less patients shared their feelings, the less they perceived life as meaningful (*p* < 0.01) (cells in orange) and the worse they felt about themselves (*p* < 0.01) (cells in brown).

On the other hand, the less the patient understands the importance of life, the worse they feel about themselves (*p* < 0.01) (cells in purple).

Finally, the greater the depressive state reported by the patients, the higher their anxiety related to the disease (*p* < 0.01) (cells in pink) and self-reported anxiety (*p* < 0.01) (cells in yellow). 

## 4. Discussion

Psychological suffering in patients with advanced diseases and those receiving palliative care is often overlooked, creating a significant gap in the holistic management of these patients. Many individuals experience fear, sadness, and grief throughout the course of their disease [13,28]. Literature indicates that psychopathological conditions such as anxiety and depression in cancer patients have prevalence rates ranging from 35% to 50% [13,29,30]. These prevalence rates can be attributed to several factors, including gender, cancer’s primary site, measurement instrument used, and the timing/setting of the evaluation [29,30].

Given the recognized impact of emotional suffering on patient well-being, its identification is essential. In the final weeks of life, patients often experience profound sadness and hopelessness, which can diminish their sense of life’s meaning and impair their ability to engage in meaningful relationships [31,32].

In our sample, the majority of participants were male, with a median age of 72 years. These findings are consistent with the existing literature. Pitzer et al. indicated that patient’s gender did not significantly affect access to palliative care, although some studies have reported worse outcomes for men [33]. Similarly, Christ et al. found that, among 337 patients admitted to a palliative care unit (inpatient care) in Switzerland during 2019, the average age was 71 years, with 56% of patients being male [34]. On the other hand, Sousa et al. examined the sociodemographic characteristics of patients admitted to a palliative care unit (inpatient care) in a Portuguese oncology hospital, finding an average age of 64 years, but, unlike our study, it was observed that 63.4% of patients were female [35].

In our sample, the most frequently observed neoplasm was digestive cancers (41.7%), followed by head and neck neoplasms (20%). These findings are consistent with some data available in the literature. For example, a study carried out in a Brazilian public hospital (inpatient care) found that the most common neoplasms were digestive (colon/rectum—22%; pancreas—16.9%; and stomach—13.6%), followed by head and neck cancers (8.5%) [36]. However, other studies have reported breast cancer as the most prevalent type of cancer in palliative care. A 2015 study found that 34% of patients had breast cancer (outpatient and inpatient care) [37].

Regarding the global Palliative Outcome Scale (POS) data, the participants exhibited a median POS score of 15 (interquartile range: 13;19) and a median Global Distress Score (GDS) of 43 (range: 17-62). A majority of the participants (70%) had a GDS ≥ 35, reflecting a high symptom burden. Furthermore, 66.6% of the participants had an Eastern Cooperative Oncology Group (ECOG) performance status of 3 or 4, indicating a significant degree of functional impairment. These results are consistent with expectations, as the primary reasons for admission to a palliative care unit are typically inadequate symptom control and significant functional limitations.

Symptom management is a fundamental pillar of palliative care. High-quality care necessitates the use of validated and effective measurement instruments to document and assess symptoms accurately.

The Edmonton Symptom Assessment System (ESAS) is a multidimensional instrument that is easy to use and demonstrates good reliability in identifying and assessing the intensity of the 10 most common symptoms in palliative care, including those in patients nearing the end of life [38]. The scale consists of ten self-report items, assessing two emotional symptoms, seven physical symptoms, and an overall sense of well-being. However, it is important to note that the emotional component of the ESAS, which includes anxiety and depression, has not been formally validated within this instrument [24,38]. According to Vignaroli et al., a score greater than 2 out of 10 on the ESAS’s anxiety and depression items in palliative care patients is considered the ideal cutoff point for screening these symptoms [24]. 

Gontijo et al. also found high prevalence rates of anxiety and depression in palliative care patients (outpatient clinic); this was associated with perceived quality of life, functional status, and the presence of other uncontrolled physical symptoms, such as fatigue, nausea, vomiting, and loss of appetite [39]. Similar to our findings, these patients had high symptom intensity (with a median GDS score of 43, ranging from 17 to 62) and low functionality (66.6% had an ECOG score of 3 or 4) [39]. Furthermore, Traldi Macedo et al. presented a cross-sectional study, involving 59 patients with advanced cancer (inpatient care), 52.5% of whom had depression and 76.3% had anxiety [36]. These data were obtained through the assessments with the ESAS tool [36]. In our study sample, the majority of patients exhibited high levels of depression and anxiety at admission to the palliative unit, with 83.3% experiencing depression and 61% experiencing anxiety.

The ESAS is a multidimensional scale that assesses symptomatic intensity, including physical and emotional symptoms. However, for a more complete assessment, it may be necessary to evaluate all dimensions of palliative care, including physical, psychological, spiritual, and social aspects.

The Palliative Outcome Scale (POS), developed by Irene Higginson at the Department of Palliative Care and Policy at King’s College London, is a 10-item multidimensional scale [40]. The scores range from 0 to 4 and assess various aspects such as pain and other physical symptoms, patient and family anxiety, emotional well-being, quality of information provided, and social support over the previous three days [40]. The POS is available in two versions: the self-version for the patient and the proxy version for healthcare staff. In this study, we utilized the self-version, as we believe that the patients’ own responses provide the most reliable information [40].

The psychological well-being component of the POS is represented by items 3, 6, 7, and 8, with the total score for these items ranging from 0 to 16 [20].

In the study, 66.7% of patients reported feeling worried and anxious about the disease process and its treatment. Only 6.7% were able to share how they were feeling, 3.4% felt that life was worthwhile, and 3.4% felt good about themselves as a person. 

Traldi Macedo et al. reported that, approximately 39% of patients (admitted to a palliative care unit—inpatient care) gave severe scores (3 or 4) on the following question: “Have you been feeling anxious or worried about your illness or treatment?” [36]. Additionally, 18.6% of patients responded similarly to the question “Have you been able to share how you are feeling with your family or friends?” [36]. Around 32% reported severe scores for the question “Have you felt that life was worthwhile?”, and 13.5% gave severe scores for the question “Have you felt good about yourself as a person?” [36].

Bausewein et al. conducted a study with 118 patients to validate the Palliative Outcome Scale (POS) in Germany [41]. The scale was administered on days 1 to 3 after admission (inpatient care), and then again after 3 or 4 days [41]. The study included all patients referred to palliative care over a 3-month period in 2002 [41]. In the initial assessment, severe scores (3/4) were reported by 41% of patients regarding anxiety about their disease or treatment [41]. Only 10% of patients reported being able to share how they felt; 36% felt that life was worthwhile; and 29% felt good about themselves as a person [41].

Antunes et al. conducted a multi-center study involving 1,703 cancer and non-cancer patients (inpatient and outpatient care) to explore the psychometric properties of the Integrated Palliative Outcome Scale (IPOS) [42]. They found that 13.3% of the participants felt anxious or worried about their disease, while 9.6% reported feeling depressed [42]. The study concluded that the IPOS is an effective tool for screening these two psychological symptoms in patients with advanced disease [42].

The discrepancies between our study and the existing literature may be attributed to several factors. The institution’s policy at the time of the study stipulated that patients would only be referred to the palliative care team once curative treatments were no longer pursued, meaning that referrals were often made at a late stage, with patients nearing the end of life. Furthermore, the study was conducted during the COVID-19 pandemic, a period marked by social isolation due to restrictions on interpersonal contact and visits from family and friends in hospitals. These factors likely contributed to a heightened sense of distress among patients. In line with this, Obispo-Portero et al. conducted a multicenter study with 401 patients with advanced cancer during the pandemic (outpatient care) and found that the incidence of anxiety and depression was 36% and 35%, respectively [43].

Lastly, the authors decided to correlate the ESAS psychological dimensions (anxiety and depression) with POS psychological questions. 

This analysis revealed several significant correlations: greater anxiety regarding the illness or treatment was associated with higher levels of self-reported anxiety (*p* < 0.01); the less the patient shares their feelings with family and friends, the less they perceive life as meaningful (*p* < 0.01), and the worse they feel about themselves (*p* < 0.05); a lower understanding of the importance of life was linked to a worse self-perception (*p* < 0.01); and higher levels of anxiety related to the disease or treatment were associated with increased depressive symptoms (*p* < 0.01).

Anxiety and concern regarding the disease and treatments measured by the POS appears to be predictive of the patient’s self-reported anxiety and depression in the ESAS. The items that assess the emotional component in the POS are self-correlated with each other, so they must be observed together.

The study demonstrates the importance of a holistic assessment and management of anxiety in palliative care. At the time this study was carried out, patients had a psychological assessment when the physician, nurse, and/or social worker identified this need and requested psychological support. This approach was adapted to the presence of only two psychologists included in the multidisciplinary team (but also with the role of supporting the entire oncology unit). This study demonstrated the importance of earlier evaluation and support of psychological symptoms, and, at the moment, the team is made up of four members, which allows for the assessment of practically all patients, especially upon admission to the unit (inpatient care). 

## 5. Limitations

The sample size is relatively small, which was influenced by the conduct of the research during the COVID-19 pandemic. 

Conducting this research in a single hospital further constrained the sample characteristics and size. Future studies should aim to expand the sample by involving multiple palliative care teams, in order to reduce bias and include a larger and more diverse patient population, including, for example, non-oncological patients. 

The cross-sectional design of the study allowed knowing the current status of the patients but did not evaluate the palliative intervention on the management of emotional symptoms. Further studies of longitudinal use of the ESAS and POS in management of distress are recommended. 

Finally, the authors would like to add that there is a long delay between the data collection and their publication, which is related to excessive work in professional terms, partly related to the COVID-19 pandemic. However, the results found make sense in the context of palliative care, as we can see in the discussion section. 

## 6. Conclusions

Emotional distress is common in patients with advanced cancer [44,45]. 

There are several tools to identify and evaluate patient with psychological symptoms and in need of more specialized assessment [46,47]. However, there is a lack of consensus regarding the best instrument to assess suffering and emotional distress in palliative care [46,47].

This study evaluated the psychometric properties of the ESAS and POS. It was found that the anxiety and concern related to the disease and treatment, as measured by the POS, served as a predictor for self-reported anxiety and depression in the ESAS. The items assessing the emotional component of the POS were strongly correlated with one another and should be used together when evaluating this dimension.

These instruments are widely used in clinical practice within palliative care due to their ease of use, which facilitates better integration into the routine operations of care teams.

Although neither of these scales is specifically designed to assess the psychological profile of patients in palliative care, they are among the most commonly utilized measurement tools by palliative care teams. Elevated scores on the psychometric items of each scale may serve as an indication for the implementation of more specific instruments, such as the Emotional Thermometer or the Anxiety and Depression Scale, and underscore the need for intervention by the team’s psychologist or psychiatrist.

## Figures and Tables

**Table 1:** Demographic and clinical characteristics of the sample, POS and ESAS scores, and ECOG performance status (*N* = 60).

Variables		Sample
Gender, *n* (%)	Male	37 (61.7)
	Female	23 (38.3)
Age (years)	Median [Q1; Q3]	72 [58.25; 79]
	Minimum	43
	Maximum	94
Education level, *n* (%)	Elementary school	49 (81.7)
	High school	5 (8.3)
	Graduation	4 (6.7)
	Absent	2 (3.3)
Marital status, *n* (%)	Married	49 (81.7)
	Single	9 (15)
	Divorced	4 (6.6)
	Widow	16 (26.7)
Residence, *n* (%)	Home	50 (83.3)
	Nursing home	10 (16.7)
Companions, *n* (%)	Spouse	30 (50)
	Son/daughter	7 (11.7)
	Siblings	1 (1.6)
	Father/mother/parents	3 (5)
	Without companion	12 (20)
	Other caregivers	7 (11.7)
Types of cancer, *n* (%)	Head and neck	12 (20)
	Cutaneous	3 (5)
	Digestive	25 (41.7)
	Gynecological	5 (8.3)
	Hematologic	1 (1.7)
	Breast	6 (10)
	Prostate	2 (3.3)
	Lung	6 (10)
Total POS Score *	Median [Q1; Q3]	15 [13; 19]
	Minimum	7
	Maximum	25
Total ESAS (GDS) Score *	Median [Q1; Q3]	42.5 [34.25;48]
	Minimum	15
	Maximum	62
ECOG performance status at admission	0	4 (6.6)
	1	6 (10)
	2	10 (16.6)
	3	25 (41.6)
	4	15 (25)

Q1—First quartile; Q3—Third quartile; * Total score was obtained by adding the items.

**Table 2:** Descriptive analysis of the ESAS Emotional and psychological dimensions by severity (*N* (%)).

	0	Mild (1–3)	Moderate (4–6)	Severe (7–10)
Depression	3 (5)	7 (11.7)	39 (65)	11 (18.3)
Anxiety *	1 (1.7)	6 (10.2)	36 (61)	16 (27.1)

* 1 missing.

**Table 3:** Descriptive analysis of participants’ responses to the psychological items of the POS scale (*N* (%)).

Items\Score	0	1	2	3	4
3. Have you been feeling ANXIETY ABOUT DISEASE or treatment?	1 (1.7)	6 (10)	13 (21.7)	36 (60)	4 (6.7)
6. Have you been able to SHARE how you are feeling? *	5 (8.5)	29 (49.2)	21 (35.6)	4 (6.8)	0 (0)
7. Have you felt that life was worthwhile (MEANING OF LIFE)?	7 (11.7)	38 (63.3)	13 (21.7)	2 (3.3)	0 (0)
8. Have you felt good about yourself as a person (SELF-FEELINGS)?	7 (11.7)	35 (58.3)	16 (26.7)	2 (3.3)	0 (0)

* 1 missing. Question 3: 0—no; 1—Occasionally; 2—Sometimes; 3—Most of the time; 4—Always. Question 6, 7, 8: 0—Always; 1—Most of the time; 2—Sometimes; 3—Occasionally; 4—Never.

**Table 4:** Spearman’s correlations between the emotional dimensions of ESAS and POS (r).

		ESAS		POS			
		Depression	Anxiety	Anxiety About Disease ^1^	Sharing Feelings ^2^	Meaning of Life ^3^	Self-Feelings ^4^
ESAS	Depression	-	0.792 **	0.386 **	0.245	0.079	0.083
	Anxiety	0.792 **	-	0.473 **	0.164	0.095	0.140
POS	Anxiety about disease ^1^	0.386**	0.473 **	-	−0.006	0.143	0.042
	Sharing feelings ^2^	0.245	0.164	−0.006	-	0.529 **	0.342 **
	Meaning of life ^3^	0.079	0.095	0.143	0.529 **	-	0.635 **
	Self-feelings ^4^	0.083	0.140	0.042	0.342 **	0.635 **	-

* *p* < 0.05; ** *p* < 0.01. 1—Have you been feeling ANXIETY ABOUT DISEASE or treatment? (Question 3). 2—Have you been able to SHARE how you are feeling? (Question 6). 3—Have you felt that life was worthwhile (MEANING OF LIFE)? (Question 7); 4—Have you felt good about yourself as a person (SELF-FEELINGS)? (Question 8). Cells in blue—correlation between self-reported anxiety and anxiety related to the disease (measured by the POS scale). Cells in orange—correlation between sharing feelings and meaning of life questions (POS). Cells in brown—correlation between sharing feelings and self-feelings questions (POS). Cells in purple—correlation between self-feelings and meaning of life questions (POS). Cells in pink—correlation between self-reported depression (ESAS) and anxiety related to the disease (POS). Cells in yellow—correlation between self-reported depression and anxiety (ESAS).

## Data Availability

The datasets generated and analyzed during the current study are available from the corresponding author upon reasonable request. Some of the results are similar to those presented in another article, as they are the result of the same research project (Gonçalves, F., Gaudêncio, M., Paiva, I., Semedo, V. A., Rego, F., & Nunes, R. (2024). Impact of Symptom Distress on the Quality of Life of Oncology Palliative Care Patients: A Portuguese Cross-Sectional Study. *Healthcare*, *12*(23), 2487. https://doi.org/10.3390/healthcare12232487).
